# Comparison of left atrial deformation parameters between renal transplant and hemodialysis patients

**DOI:** 10.1186/s12947-022-00275-4

**Published:** 2022-02-25

**Authors:** Ufuk Yildirim, Murat Akcay, Metin Coksevim, Ercan Turkmen, Okan Gulel

**Affiliations:** 1 Department of Cardiology, Faculty of Medicine, Ondokuz Mayis University, Samsun, Turkey; 2 Department of Nephrology, Faculty of Medicine, Ondokuz Mayis University, Samsun, Turkey

**Keywords:** Renal transplantation, Hemodialysis, Speckle tracking echocardiography, Left atrial function, Strain, Strain rate

## Abstract

**Background:**

Renal transplantation (RT) has been demonstrated to improve left ventricular systolic function. However, only few studies have attempted to reveal the effects of transplantation on left atrial (LA) function. In our study, we aimed to compare LA function between RT and hemodialysis patients.

**Methods:**

This cross-sectional study included 75 consecutive patients with RT, and 75 age- and gender-matched patients on maintenance hemodialysis. LA strain and strain rate (SR) analyzed by two-dimensional (2D) speckle tracking echocardiography (STE) were compared between the groups in addition to standard echocardiographic parameters.

**Results:**

LA strain during reservoir phase (29.88 ± 5.76% vs 26.11 ± 5.74%, *P* < .001), LA strain during conduit phase (− 15.28 ± 5.00% vs − 12.92 ± 4.38%, *P* = .003), and LA strain during contraction phase (− 14.60 ± 3.32% vs − 13.19 ± 3.95%, *P* = .020) were higher in the transplantation group. Similarly, LA peak SR during reservoir phase (1.54 ± 0.33 s^− 1^ vs 1.32 ± 0.33 s^− 1^, *P* < .001), LA peak SR during conduit phase (− 1.47 ± 0.49 s^− 1^ vs − 1.12 ± 0.42 s^− 1^, *P* < .001), and LA peak SR during contraction phase (− 2.13 ± 0.46 s^− 1^ vs − 1.83 ± 0.58 s^− 1^, *P* = .001) were higher in the transplantation group as well.

**Conclusions:**

LA function assessed by 2D STE was better in RT patients than hemodialysis patients. This may suggest favorable effects of RT on LA function.

## Introduction

It is well established that chronic kidney disease (CKD) is associated with important cardiac alterations such as left ventricular (LV) hypertrophy, LV systolic and diastolic dysfunction, LV and left atrial (LA) dilatation [1, 2]. Even though structural cardiac alterations are initially adaptive, they may progress over time and lead to cardiac dysfunction [1]. Renal transplantation (RT) has reduced the risk of CV death compared with long-term dialysis [3]. In addition, LV systolic function tends to improve after RT in patients with end-stage renal disease (ESRD) [1, 4, 5]. However, only few studies have attempted to reveal the effects of RT on LA function.

Two-dimensional (2D) speckle tracking echocardiography (STE) has been well validated as a quantitative tool to evaluate LV function [6]. This modality has also been applied to assess LA function. During the cardiac cycle, LA has three main functions: reservoir function in systole when blood fills LA, conduit function in early diastole corresponding to passive LV filling, and active contractile function in late diastole [7]. LA deformation parameters analyzed by STE provide a window on all phases of LA function [8]. CKD is an independent factor affecting LA function [9]. In our study, we aimed to compare LA deformation parameters between RT and hemodialysis patients.

## Methods

### Study population

A total of 150 patients with CKD followed up at the Nephrology Outpatient Clinic were included into this cross-sectional study, and two groups were formed. Seventy-five consecutive patients with RT having a functional graft > 1 year and estimated glomerular filtration rate (eGFR) > 60 mL/min/1.73 m^2^ were compared with 75 age- and gender-matched patients receiving hemodialysis three times per week. Modification of Diet in Renal Disease formula was used to calculate eGFR.

Exclusion criteria were as follows: age < 18 years or > 65 years, body mass index ≥40 kg/m^2^, clinical symptoms and signs of hypervolemia, LV ejection fraction (LVEF) < 45%, documented ischemic heart disease, moderate to severe heart valve disease or history of heart valve surgery, hypertrophic or restrictive cardiomyopathy, constrictive pericarditis, atrial fibrillation, QRS duration ≥120 ms, history of RT rejection, and poor echocardiographic image quality.

The study was approved by the institutional ethics committee. Informed consent was obtained from all participants included in the study.

### Echocardiography

Echocardiography was performed using a Vivid E9 echocardiography machine (GE Vingmed Ultrasound, Horten, Norway) and M5S ultrasound probe (1.5–4.5 MHz). All echocardiograhic examinations were made by the same physician at midday to avoid circadian changes. For the patients on maintenance hemodialysis, echocardiographic measurements were performed on the next day after dialysis treatment [10].

Cardiac chamber dimensions were measured in accordance with the recommendations of the American Society of Echocardiography and the European Association of Cardiovascular Imaging [11]. Linear internal dimensions of LV and its walls were acquired in the parasternal long-axis view and measured at the level of the mitral valve leaflet tips directly from 2D echocardiographic images. LV mass was calculated using the Devereux’s formula and indexed to body surface area (BSA). LVEF was estimated using modified Simpson method. LA anteroposterior diameter (LAAPD) was obtained in the parasternal long-axis view and measured at the level of the aortic sinuses from 2D echocardiographic images. LA volume was calculated using biplane area-length method and indexed to BSA. Pulmonary artery systolic pressure (PASP) was estimated by using the formula based on tricuspid regurgitant jet velocity, inferior vena cava diameter and collapsibility. Pulsed-wave (PW) Doppler was performed between mitral leaflet tips in the apical 4-chamber view to acquire mitral inflow velocities and PW tissue Doppler imaging was performed at the septal and lateral border of mitral annulus in the apical 4-chamber view to acquire average mitral annular velocities. Peak velocity of early mitral inflow (mitral E), peak velocity of late mitral inflow (mitral A), ratio of mitral E to mitral A (mitral E/A), peak systolic velocity of mitral annulus (mitral s’), peak early diastolic velocity of mitral annulus (mitral e’), peak late diastolic velocity of mitral annulus (mitral a’), and ratio of mitral E to mitral e’ (mitral E/e’) were obtained.

### Two-dimensional speckle tracking analysis of left atrium

**Fig. 1:**
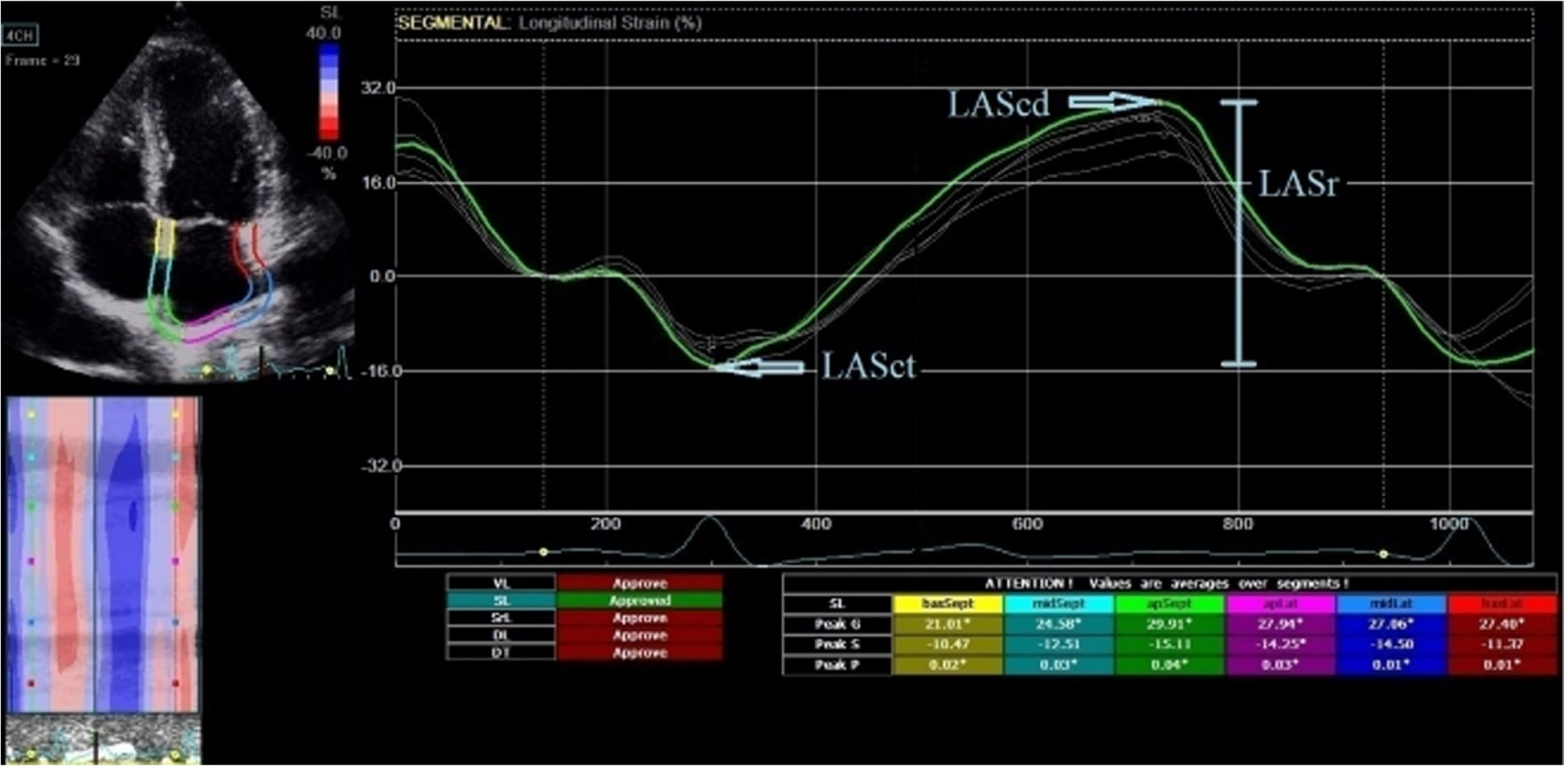
Left atrial strain curves. LASr, left atrial strain during reservoir phase; LAScd, left atrial strain during conduit phase; LASct, left atrial strain during contraction phase

**Fig. 2:**
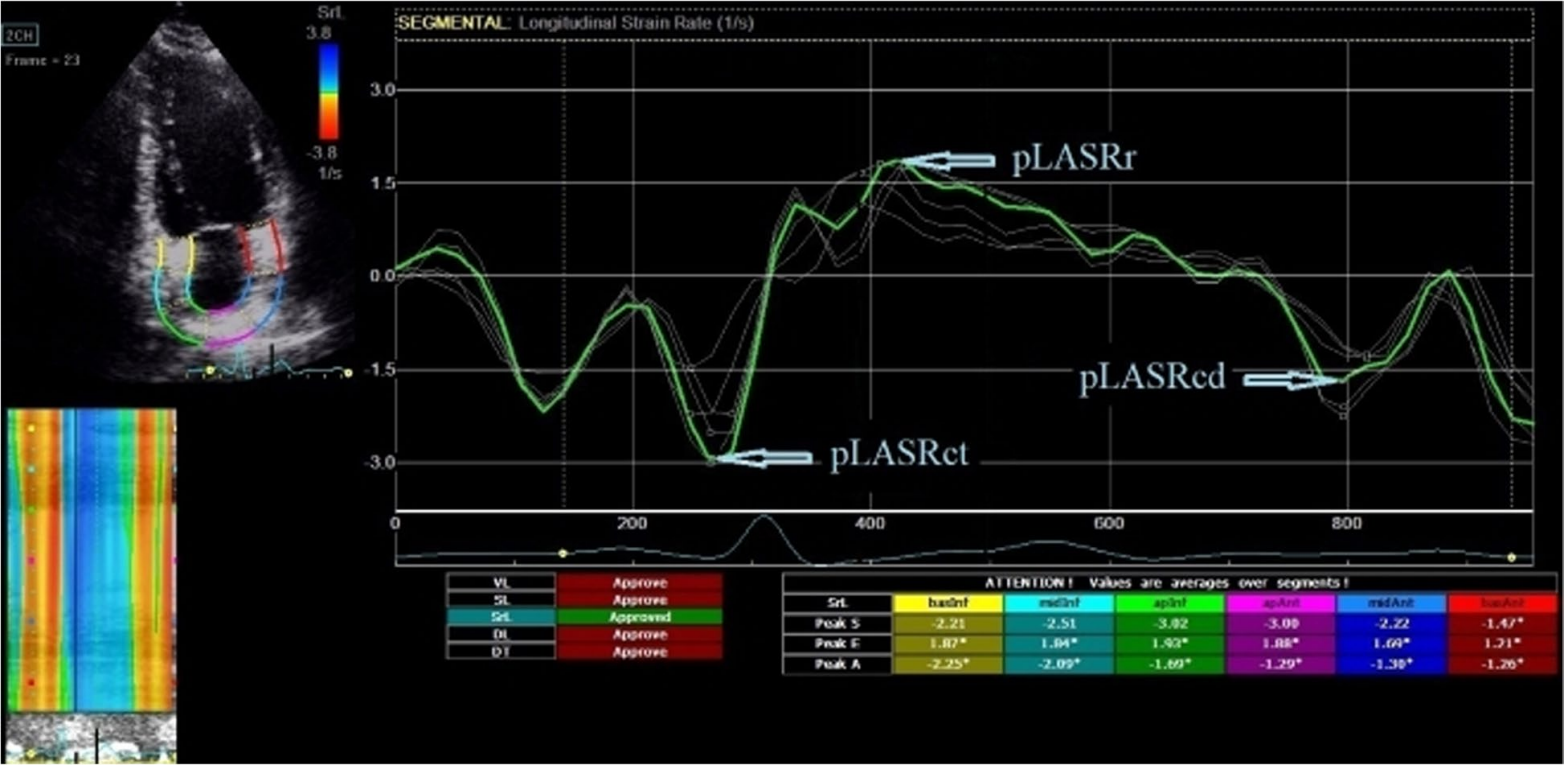
Left atrial strain rate curves. pLASRr, left atrial peak strain rate during reservoir phase; pLASRcd, left atrial peak strain rate during conduit phase; pLASRct, left atrial peak strain rate during contraction phase

For 2D speckle tracking analysis of LA, apical 4-chamber and 2-chamber view images were acquired at a frame rate of 60–80 frames/s. Three consecutive cardiac cycles were recorded in each view for offline analysis using the software (EchoPac PC, version 110.1.2, GE Vingmed Ultrasound, Horten, Norway). LA deformation parameters were analyzed by another physician blinded to the patient’s clinical status. We used P wave onset of the electrocardiogram as the reference point to calculate LA strain and strain rate (SR), as previously recommended in sinus rhythm [12]. Using P wave as the reference point enabled to identify LA strain during contraction phase (LASct), LA strain during conduit phase (LAScd), and LA strain during reservoir phase (LASr) (Fig. 1). Similarly, we specified LA peak SR during contraction phase (pLASRct), LA peak SR during reservoir phase (pLASRr), and LA peak SR during conduit phase (pLASRcd) (Fig. 2).

A spesific cardiac cycle was selected for each view, and LA endocardial surface was manually traced in both apical 4-chamber and 2-chamber view images by a point-and-click approach. Epicardial surface tracing was automatically created by the system, forming a region of interest (ROI), which could be manually adjusted in width and shape. ROI was divided into 6 segments by the software in each view. Segments without adequate image quality for speckle tracking analysis were excluded from the evaluation by the system. The average values of LA deformation parameters for each apical view were calculated, and the final values were the averages of the ones calculated for each apical view.

### Intraobserver and interobserver variability

Intraobserver and interobserver variability for LA deformation parameters were evaluated by re-analyzing the recorded images of 12 randomly selected patients with 6 subjects from each group. Speckle tracking analyzes were repeated by the same individual after at least 1 week for intraobserver variability and by another physician for interobserver variability.

### Statistical analysis

The research data were uploaded and evaluated by using IBM Statistical Package for Social Sciences version 25. Descriptive statistics for categorical variables were reported as frequencies and percentages. Pearson Chi Square was used to compare categorical variables in addition to descriptive statistics. The suitability of numerical variables with normal distribution was determined by visual (histograms and probability plots) and analytical (Kolmogorov-Smirnov and Shapiro-Wilk tests) methods. Descriptive statistics for numerical variables with normal distribution were reported as mean ± standard deviation. Independent Samples t test was used for the variables with normal distribution to identify significant differences between the groups and Levene’s test was applied to determine homogeneity of variances. Descriptive statistics for numerical variables without normal distribution were reported as median (minimum-maximum). Mann-Whitney U test was used for comparisons of variables without normal distribution. When comparing LA deformation parameters between RT and hemodialysis patients in Table 3, analysis of covariance (ANCOVA) was also performed to control the effects of systolic blood pressure (SBP), serum hemoglobin level, and LVEF on the outcomes. Pearson correlation coefficient was used to measure the magnitude and direction of the relationships between numerical variables with normal distribution, and Spearman correlation coefficient was used for numerical variables without normal distribution. R ≥ 0.7 was defined as strong relationship, 0.5 ≤ R < 0.7 was defined as moderate relationship, and 0.3 ≤ R < 0.5 was defined as weak relationship. Intraobserver and interobserver variability were assessed using intraclass correlation coefficient (ICC) by Cronbach. Statistical significance level was accepted as *P* < 0.05.

## Results

### Baseline characteristics of the study population

Baseline characteristics of the study population are presented in Table 1. There was no significant difference between the groups in terms of age, gender, diastolic blood pressure (DBP), heart rate, history of hypertension, duration of hypertension, history of diabetes mellitus, and duration of diabetes mellitus. SBP was significantly higher in the hemodialysis group (*P* < .001). Median time of CKD duration was longer in the transplantation group (*P* = .015). Median time of CKD duration before RT was 5 (1–19) years, median time of dialysis treatment before RT was 3 (1–15) years, and median time after RT was 3 (1–12) years in the transplantation group. Median time after the initation of dialysis treatment was 3 (1–20) years in the hemodialysis group. Serum hemoglobin level was significantly higher in the transplantation group (*P* < .001).

**Table 1:** Baseline characteristics of the study population

Variable	Transplantation group	Hemodialysis group	***P***
Age (year)	41.3 ± 10.5	43.3 ± 10.6	.257
Male (%^a^)	39 (52.0)	37 (49.3)	.870
SBP (mmHg)	123.5 ± 15.0	133.2 ± 17.5	**<.001**
DBP (mmHg)	78.2 ± 9.2	80.4 ± 10.2	.167
Heart rate (beats/min)	80.3 ± 11.7	79.3 ± 13.1	.637
History of hypertension (%^a^)	58 (77.3)	64 (85.3)	.295
Duration of hypertension (year)	8 (1–20)	8 (1–25)	.429
History of diabetes mellitus (%^a^)	13 (17.3)	14 (18.7)	.832
Duration of diabetes mellitus (year)	9.0 ± 5.9	12.7 ± 6.8	.142
Duration of CKD (year)	10 (3–25)	7 (1–24)	**.015**
Serum hemoglobin level (g/dL)	13.71 ± 1.84	12.02 ± 1.50	**<.001**

^a^Column percentage

### Standard echocardiographic parameters

LV diastolic diameter (LVDD), LV systolic diameter (LVSD), septal wall thickness (SWT), posterior wall thickness (PWT), and LV mass index (LVMi) were significantly lower in the transplantation group compared to the hemodialysis group (*P* = .004, *P* < .001, *P* = .001, *P* = .001, and *P* < .001, respectively). LVEF was significantly higher in the transplantation group (*P* < .001). LAAPD and LA volume index (LAVi) were significantly lower in the transplantation group than the hemodialysis group (*P* < .001 for each) (Table 2).

**Table 2:** Standard echocardiographic parameters

Variable	Transplantation group	Hemodialysis group	***P***
LVDD (mm)	43 (36–58)	48 (34–58)	**.004**
LVSD (mm)	26 (19–43)	32 (19–45)	**<.001**
SWT (mm)	12 (7–17)	13 (7–17)	**.001**
PWT (mm)	10 (7–15)	12 (7–17)	**.001**
LVMi (g/m^2^)	96.8 ± 25.2	130.5 ± 41.2	**<.001**
LVEF (%)	66.6 ± 5.7	59.8 ± 6.5	**<.001**
LAAPD (mm)	33 (26–45)	37 (25–45)	**<.001**
LAVi (mL/m^2^)	23 (13–55)	34 (13–60)	**<.001**
Mitral E (cm/s)	83.7 ± 19.8	88.3 ± 25.1	.219
Mitral A (cm/s)	79.9 ± 16.6	90.0 ± 18.0	**<.001**
Mitral E/A	1.1 (0.5–1.6)	0.9 (0.5–1.6)	.070
Mitral s’ (cm/s)	9 (6–15)	7 (5–12)	**<.001**
Mitral e’ (cm/s)	10.1 ± 3.0	8.4 ± 2.7	**.001**
Mitral a’ (cm/s)	10 (7–18)	9 (5–17)	**.003**
Mitral E/e’	8.1 (4.7–18.0)	10.8 (4.2–24.5)	**<.001**
PASP (mmHg)	26.9 ± 6.8	28.3 ± 7.7	.229

Mitral E, mitral E/A, and PASP did not significantly differ between the groups. Mitral A and mitral E/e’ were significantly lower in the transplantation group compared to the hemodialysis group (*P* < .001 for each). Mitral s’, mitral e’, and mitral a’ were significantly higher in the transplantation group (*P* < .001, *P* = .001, and *P* = .003, respectively) (Table 2).

### Left atrial deformation parameters

LASr, LAScd, and LASct were significantly higher in the transplantation group compared to the hemodialysis group (*P* < .001, *P* = .003, and *P* = .020, respectively). Similarly, pLASRr, pLASRcd, and pLASRct were significantly higher in the transplantation group (*P* < .001, *P* < .001, and *P* = .001, respectively) as well. After adjusting for SBP, serum hemoglobin level, and LVEF, statistical significance was preserved except for LASct and pLASRct (*P* = .274 and *P* = .076, respectively) (Table 3).

**Table 3:** Left atrial deformation parameters

Variable	Transplantation group	Hemodialysis group	***P***	P*
LASr (%)	29.88 ± 5.76	26.11 ± 5.74	**<.001**	**.014**
LAScd (%)	−15.28 ± 5.00	−12.92 ± 4.38	**.003**	**.030**
LASct (%)	− 14.60 ± 3.32	−13.19 ± 3.95	**.020**	.274
pLASRr (s^−1^)	1.54 ± 0.33	1.32 ± 0.33	**<.001**	**.006**
pLASRcd (s^−1^)	− 1.47 ± 0.49	−1.12 ± 0.42	**<.001**	**.001**
pLASRct (s^−1^)	−2.13 ± 0.46	−1.83 ± 0.58	**.001**	.076

^*^After adjustment for SBP, serum hemoglobin level, and LVEF

When considering only strong and moderate relationships; LASr had a positive moderate correlation with mitral e’ (R = .608, *P* < .001), LAScd had a positive moderate correlation with mitral e’ (R = .674, *P* < .001), pLASRcd had a positive strong correlation with mitral e’ (R = .766, *P* < .001) and a negative moderate correlation with PWT (R = −.506, *P* < .001). Many more significant but weak relationships were also detected between LA deformation parameters and standard echocardiographic parameters (Table 4).

**Table 4:** Correlation analysis

Variable	LASr		LAScd		LASct		pLASRr		pLASRcd		pLASRct	
	R	***P***	R	***P***	R	***P***	R	***P***	R	***P***	R	***P***
LVDD	−.139	.093	−.002	.977	−.269	**.001**	−.187	**.023**	−.164	**.047**	−.260	**.001**
LVSD	−.238	**.004**	−.085	.304	−.328	**<.001**	−.197	**.016**	−.215	**.009**	−.349	**<.001**
SWT	−.241	**.003**	−.294	**<.001**	.002	.984	−.227	**.005**	−.418	**<.001**	−.117	.158
PWT	−.333	**<.001**	−.345	**<.001**	−.099	.230	−.312	**<.001**	−.506	**<.001**	−.233	**.004**
LVMi	−.315	**<.001**	−.230	**.005**	−.214	**.009**	−.357	**<.001**	−.402	**<.001**	−.270	**.001**
LVEF	.262	**.001**	.123	.138	.309	**<.001**	.160	.052	.179	**.029**	.326	**<.001**
LAAPD	−.324	**<.001**	−.214	**.009**	−.282	**.001**	−.322	**<.001**	−.419	**<.001**	−.362	**<.001**
LAVi	−.327	**<.001**	−.212	**.010**	−.292	**<.001**	−.357	**<.001**	−.404	**<.001**	−.421	**<.001**
Mitral E	.154	.062	.294	**<.001**	−.213	**.009**	−.120	.146	.208	**.011**	−.204	**.013**
Mitral A	.009	.913	−.109	.187	.174	**.034**	.053	.525	−.238	**.004**	.056	.500
Mitral E/A	.119	.149	.355	**<.001**	−.337	**<.001**	−.158	.056	.366	**<.001**	−.237	**.004**
Mitral s’	.369	**<.001**	.371	**<.001**	.130	.116	.486	**<.001**	.464	**<.001**	.336	**<.001**
Mitral e’	.608	**<.001**	.674	**<.001**	.112	.176	.418	**<.001**	.766	**<.001**	.313	**<.001**
Mitral a’	.162	**.049**	−.072	.382	.413	**<.001**	.408	**<.001**	−.049	.554	.459	**<.001**
Mitral E/e’	−.413	**<.001**	−.390	**<.001**	−.172	**.037**	−.451	**<.001**	−.498	**<.001**	−.395	**<.001**
PASP	−.090	.276	−.037	.653	−.098	.235	−.253	**.002**	−.121	.144	−.137	.097

ICC analysis of intraobserver and interobserver variations for LA deformation parameters is presented in Table 5.

**Table 5:** Intraobserver and interobserver variability

Variable	Intraobserver variation			Interobserver variation		
	ICC	95% CI	***P***	ICC	95% CI	***P***
LASr	0.987	0.955–0.996	**<.001**	0.985	0.947–0.996	**<.001**
LAScd	0.978	0.924–0.994	**<.001**	0.960	0.862–0.989	**<.001**
LASct	0.966	0.882–0.990	**<.001**	0.973	0.905–0.992	**<.001**
pLASRr	0.938	0.786–0.982	**<.001**	0.893	0.628–0.969	**<.001**
pLASRcd	0.991	0.969–0.997	**<.001**	0.979	0.928–0.994	**<.001**
pLASRct	0.957	0.852–0.988	**<.001**	0.970	0.895–0.991	**<.001**

*CI* Confidence interval

## Discussion

In the present study, the comparison between RT and hemodialysis patients revealed that LASr, LAScd, and LASct were significantly higher in patients with RT than patients on maintenance hemodialysis. We also demonstrated that pLASRr, pLASRcd, and pLASRct were significantly higher in RT patients as well. LVEF and serum hemoglobin level were significantly higher, SBP was significantly lower in RT recipients in accordance with the literature [4]. Nevertheless, in order to understand whether the results are just the reflection of higher LVEF in the transplantation group and to control the possible effects of serum hemoglobin level and SBP on LA deformation parameters, adjusted *P* values were obtained. After adjusting for LVEF, serum hemoglobin level, and SBP, statistical significance was preserved for parameters indicating LA reservoir and conduit function, but no longer present for parameters indicating LA contractile function. In addition, we detected many significant relationships between LA deformation parameters and standard echocardiographic parameters, one of which was the strong correlation between mitral e’ and pLASRcd.

LA size and function are important predictors of CV morbidity and mortality in patients with CKD [13]. During the cardiac cycle, LA has three main functions. Reservoir function corresponding to LV isovolumic contraction, ejection, and isovolumic relaxation is influenced by LV systolic function, atrial size and compliance. Conduit function corresponding to early transmitral flow is influenced by atrial compliance, LV relaxation and compliance. Lastly, contractile function corresponding to late transmitral flow is influenced by atrial contractility, atrial preload, atrial afterload, and LV systolic reserve [14]. The alterations of LA size and function in patients with CKD are multifactorial. LV hypertrophy, LV diastolic dysfunction, and volume overload may cause increased LV filling pressure and LA afterload in these patients [13]. These alterations prompt a compensatory mechanism in LA characterised by LA dilatation [15]. Late stage chronic increases in LA afterload in addition to LA remodelling may prompt the alterations in the compliance, reservoir function, and contractile function of LA [10]. LA remodelling is also associated with atrial interstitial fibrosis and cell hypertrophy, which may contribute to impaired LA function [16]. It has been demonstrated that LA deformation parameters assessed by STE provide a better diagnostic performance in indicating subclinic LA dysfunction in patients with CKD as compared to the Doppler parameters which have been shown to be very preload-dependent [17, 18]. Even though LAVi is less affected by preload [19], it cannot accurately represent LA function [20]. The abnormalities in LA deformation parameters are relatively independent from LA dilatation and volume overload [21], so the alterations in LA strain and SR may precede the alterations in LA volume [22]. Kadappu et al. demonstrated that LA strain was the most significant and sensitive parameter to detect myocardial involvement in patients with CKD [23].

Few studies have attempted to reveal the effects of RT and hemodialysis on LA size and function. It has been reported that there may be a reduction in LA size after RT in patients with ESRD [24, 25]. However, in the study of Hewing et al. evaluating 31 RT recipients by echocardiography before and after RT with a median follow-up of 19 months, LAVi and LA strain analyzed by STE did not change significantly after RT. [1] In our study in which median time after RT was 3 years in the transplantation group, RT recipients had better outcomes in terms of LAVi and LA deformation parameters compared to hemodialysis patients. Successful RT in patients with uremic cardiomyopathy initiates the process of LA recovery in long-term clinical observation [26]. It is obvious that further studies are required to clarify this issue.

It has been reported that patients with ESRD have altered LA function [15, 17, 27] and LA strain values tend to decrease as systolic and diastolic LV function deteriorate [28]. ESRD is a complex metabolic disorder that can cause cardiac structural and functional changes known as uremic cardiomyopathy. Long-term exposure to uremic toxins leads to fibrosis and death of myocytes in addition to potentially negative inotropic and chronotropic effects [29]. With elimination of uremic environment after successful RT, uremia-specific CV risk factors such as inflammation, oxidative stress, endothelial dysfunction, anemia, abnormal calcium-phosphorous metabolism, and secondary hyperparathyroidism can improve in patients with ESRD [30]. These patients may have an improvement in blood pressure, LV mass, and LV systolic function after RT. [1, 4] On the other hand, increased oxidative stress and sympathetic activation, impaired endothelial function, and volume load between hemodialysis sessions are some of the factors which increase the likelihood of CV disease in hemodialysis patients [31]. These factors may be associated with the different effects of RT and hemodialysis on LV and LA function.

In the present study, further differences in addition to LA deformation parameters were detected between the groups. SBP was lower in the transplantation group. It has been reported that patients with ESRD may experience an improvement in SBP after successful RT. [1] Consistent with the literature [4], RT recipients in our study had higher serum hemoglobin level compared to patients on maintenance hemodialysis. LVMi was also lower in patients with RT, in line with the previous studies [1, 4, 32, 33]. LV systolic function tends to improve after RT. [1, 4, 5] Despite longer CKD duration, RT recipients had higher LVEF in the present study. In a study comparing Doppler and tissue Doppler echocardiographic parameters between 30 patients with RT and 30 patients with ESRD; mitral E/A, s’, e’, a’ tended to be higher in patients with RT, but mitral A tended to be higher in patients with ESRD [34]. Similarly, in our study comparing 75 patients with RT and 75 patients on maintenance hemodialysis; mitral s’, e’, a’ were significantly higher in the transplantation group, mitral E/A tended to be higher in the transplantation group (*P* = .070), mitral A was significantly higher in the hemodialysis group, and mitral E/e’ was also significantly higher in the hemodialysis group.

Our findings imply that successful RT may be associated with an improvement in LA deformation parameters which have been demonstrated to be the best predictor of CV outcomes in patients with CKD [35]. Among the standard echocardiographic parameters, mitral e’ shows the best correlation with LA deformation parameters, particularly with the ones indicating LA reservoir and conduit function. In addition, RT may lead to an improvement not only in LV systolic function but also in LV diastolic function.

To the best of our knowledge, this is the first study to present data regarding LA SR values in patients undergoing RT. SR has been shown to be less affected by loading conditions compared to strain [36]. Therefore, evaluation of LA SR may provide additional information for the assessment of LA function in patients with CKD. Moreover, this study reveals the most comprehensive correlation analysis between LA deformation parameters and standard echocardiographic parameters.

The present study has also some limitations. This cross-sectional study was not a follow-up study. We did not have echocardiographic data of the patients before the initiation of hemodialysis and RT. Even though the sample size of the study was relatively small, significant differences were observed between the groups. Patients with clinical symptoms and signs of hypervolemia were excluded, but invasive measurements were not performed for the assessment of volume status.

## Conclusions

LA function assessed by 2D STE was better in patients with RT than patients on maintenance hemodialysis. This may suggest favorable effects of RT on LA function.

## Data Availability

The datasets used and/or analysed during the current study are available from the corresponding author on reasonable request.
